# Soil C and N dynamics and hydrological processes in a maize-wheat rotation field subjected to different tillage and straw management practices

**DOI:** 10.1016/j.agee.2019.106616

**Published:** 2019-12-01

**Authors:** Li Wang, Xiaoliang Yuan, Chuang Liu, Zhiguo Li, Fang Chen, Shiqing Li, Lianhai Wu, Yi Liu

**Affiliations:** a Key Laboratory of Aquatic Botany and Watershed Ecology, Wuhan Botanical Garden, Chinese Academy of Sciences, Wuhan 430074, China; b Center of Conservation Biology/Economic Botany/Plant Ecology, Core Botanical Gardens, Chinese Academy of Sciences, Wuhan 430074, China; c State Key Laboratory of Soil Erosion and Dryland Farming on the Loess Plateau, Institute of Soil and Water Conservation, Chinese Academy of Sciences and Ministry of Water Resource, Yangling 712100, China; d Sustainable Agricultural Sciences, Rothamsted Research, North Wyke, Okehampton EX20 2SB, UK

**Keywords:** Danjiangkou Reservoir, Conservation tillage, SPACSYS, Surface runoff, Soil C and N balances

## Abstract

The impact of farmland nutrient losses on environment security is of serious concern. Conservation tillage led to reduced water and soil losses and increased grain yield, and is therefore one potential solution, but this approach requires an understanding of the complex adaptive traits for environment conditions. In this study, a 4-year field experiment was conducted to quantify the crop yield, runoff and soil water, organic C and N content dynamics in summer maize-winter wheat rations subjected to different tillage and straw management practices. Based on these, the effects of different tillage and straw management regimes on water, C and N balances of the soil-plant system was evaluated with a 11-year model prediction using the SPACSYS model. The treatments used in this study included conventional tillage (CT) with straw removal, conventional tillage with straw returning (CTSR), reduced tillage (RT) with straw removal and reduced tillage with straw returning (RTSR). The results showed that maize yield was remarkably affected by straw returning while there was no significant tillage effect. By contrast, wheat yield showed a high inter-annual variability, but was not significantly influenced by tillage and straw management practices. The soil water balance analysis demonstrated that the treatments with straw returning improved water use efficiency by increasing transpiration while reducing water losses through evaporation and runoff, compared to the straw-removal treatments. The simulations for all of the treatments showed that the soils acted as C and N sinks in the present study. Furthermore, plots that included straw returning amassed more C and N in the soil than the that with straw removal. Our work demonstrates that in maize-wheat rotation slopping land reduced tillage with straw returning is a win-win practice for the equilibrium between agricultural productivity and low soil water, C and N losses.

## Highlights

• Maize yield was enhanced by straw returning while there was no significant tillage effect.
• Both reduced tillage and straw returning significantly decreased runoff.
• Straw returning reduced soil water evaporation but increased water transpiration.
• Soils subjected to all the treatments acted as C and N sinks in this study.

## 1 Introduction

The impact of farmland nutrient losses on environment security is of serious concern (Chen et al., 2011; Ti et al., 2019). Agriculture management practices, such as conservation tillage and rational fertilization, led to reduce water and soil losses and increase grain yield are potential solution (Dikgwatlhe et al., 2014; Xia et al., 2016), but these approaches require an understanding of complex adaptive traits for climatic factors and environment conditions (Zhang et al., 2018). Soil carbon (C) and nitrogen (N) cycling and hydrological processes – the main ecosystem components studied by ecologists and global change scientists – play a key role in agro-ecological systems and can both positively and negatively affect crop production and soil quality (Chen and Coops, 2009; Xia et al., 2016). Thus, the actual impacts of conservation tillage practices on these processes need to be clarified if we want to simultaneously increase crop production and reduce soil nutrient and water losses (Zhao et al., 2012).

Conservation tillage including crop straw returning and reducing tillage intensity, is a new approach that has been suggested to benefit agriculture by increasing crop yields, conserving soil water and reducing seasonal evaporation; in this way, conservation tillage supports sustainable agricultural development (Zhang et al., 2007, 2018). Previous research has shown that straw returning enhances organic C sequestration and N levels in soil, and is particularly relevant for reducing soil nutrient losses (e.g. through run-off and gas emissions) and improving soil physical properties (Dikgwatlhe et al., 2014). Along with improved soil nutrient contents, several researchers have noted that straw returning to the soil can significantly improve soil moisture by benefiting both infiltration and soil water retention and reducing evaporation from the soil surface (Tan et al., 2002). Thus, it has been widely reported that crop straw returning benefits both soil fertility and crop production (Kurothe et al., 2014).

Tillage can also have beneficial effects on crop production. For instance, tillage before a new crop is planted or sown can improve crop growth and development according to several studies (Wang et al., 2002; Ogle et al., 2012). However, excessive tillage intensity is very far from being sustainable and environmentally compatible, and intensive ploughing may result in decreases in soil fertility, water availability and eventually declining crop production. These detrimental effects can be modified to some extent through reduced or zero tillage. A synthetic analysis highlighted that reduced tillage with straw returning, when compared with conventional or intensive tillage on slopes, could decrease soil loss and runoff, with the observed reduction ranging from 21.9 to 27.2% (Sun et al., 2015). However, the implementation of a no-tillage system can occasionally decrease crop yield, and this finding can be explained by poor seeding caused by previous crop residues on the soil surface (Liu et al., 2010), or by high occurrence of weeds (Buhler, 1995).

It is well known that soil water, C and N processes have very close interactions in soil and plant systems (Rhymes et al., 2016). Soil moisture, for example, can significantly affect plant growth and ecosystem productivity (Haghverdi et al., 2017), microbial activities (Singh et al., 2011), and greenhouse gas (GHG) emissions (Kumar et al., 2016). Moreover, in agro-ecosystems, C fixation by crops is closely coupled to water loss, which mainly occurs via leaf stomata. However, monitoring soil C and N cycles and water fluxes in agricultural systems is both time-consuming and expensive (Bonachela et al., 2018). For this reason, model simulations of the soil-plant-atmosphere continuum are effective for understanding C and N dynamics in terms of transformations in soil, ﬂows to water and losses to air (Li et al., 2017a). Considerable efforts have been made to predict and describe the C, N and water cycles at the soil-plant-atmosphere continuum using computer models (Huang et al., 2016; Liu et al., 2018a). SPACSYS (Soil-Plant-Atmosphere Continuum System) is a process-based, dynamic model that can simulate plant growth and development, soil N and C fluxes, soil water movement and heat transformation at the field-scale (Wu et al., 2007). The model has been used to estimate GHG emissions, describe soil C and N stocks, and predict crop yield (Perego et al., 2016; Li et al., 2017a, 2018a).The model enable us to select appropriate crop and field management practices to capture water and radiation resources and reduce the adverse impact on environment (Wu et al., 2016).

**Fig. 1:**
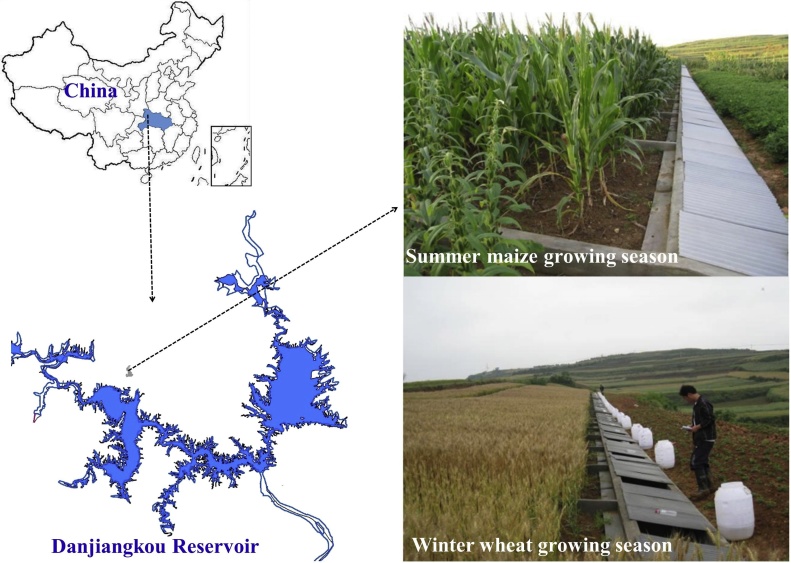
Location of the study area within China, together with photographs showing runoff plots under the summer maize and winter wheat growing seasons.

The Danjiangkou Reservoir (Fig. 1) that covers a surface area of approximately 840 km^2^ was established in 1973 as one of the water resource areas for China's South-to-North Water Diversion Project. This region includes rocky mountainous and hilly areas and is characterized by a fragile ecological environment (Liu et al., 2018b). Economic and societal development in the area has resulted in deterioration of the ecosystems and water quality of some watershed tributaries and the reservoir area. Research has reported that land management practices such as deforestation and intensive tillage - which cause soil erosion, water pollution and soil C losses – continue to degrade soil quality in the area (Liu et al., 2012). Thus, local stakeholders are interested in developing sustainable land management practices that focus on other ecosystem services than just maximizing crop yield (Li et al., 2017b). Following government-mandated land reorganization, popular agricultural management practices have been converted to more sustainable practices (e.g., reduced or no tillage, crop residue management and rational application of fertilizer) to shift towards protection, conservation, and the improvement of soil and water resources (Dou et al., 2016). To date, only a few studies have quantitatively assessed C and N cycling and water processes in a summer maize-winter wheat rotation system involving different tillage and straw management practices in this region. By combining experimental field observations with model predictions, this study aims to, 1) quantify the yield, runoff and soil water, organic C and N contents dynamic under different tillage and straw management practices, and 2) evaluate how different tillage and straw management regimes affect water, C and N balances of the soil-plant system using the SPACSYS model.

## 2 Materials and methods

### 2.1 Field site and experimental design

The studied site was located in the Xiaofuling watershed (32.763 °N, 111.161 °E, ca. 248 m above sea level) of the Danjiangkou Reservoir area, China (Fig. 1), which has a subtropical monsoon climate. The average annual precipitation between 1990 and 2018 was 750 ± 70 mm, with 70–80% of the precipitation falling between April and October. The annual average temperature for the same period was 15.7 ± 2.3 °C, with 250 ± 15 frost-free days and average annual sunshine of around 1950 ± 143 h. Soil in the area is classified as Eustric Planosols (FAO, 1974), developed from yellow cinnamon soil (Gong et al., 2007) (Table 1). Wheat, maize and rice are the staple crops across most of the region. Winter wheat is usually rotated with summer maize in the uplands and with rice in the paddy fields.

**Table 1:** Soil physical and chemical properties (0–20 cm) at the start of the field experiment in 2008.

Property	Value	Unit
Soil classification (FAO)	Eustric Planosols (FAO)	
Lay content (<2 μm)	37	%
Silt content (2–50 μm)	59	%
Sand content (50–2000 μm)	4	%
Bulk density	1.3	g cm^−3^
Soil pH (1:1 w/v water)	6.5	
Soil organic matter (SOM)	10.8	g kg^−1^
Total nitrogen (TN)	0.8	g kg^−1^
Available phosphorus (AP)	13.7	mg kg^−1^
Available potassium (AK)	125.6	mg kg^−1^

The field experiment was conducted in a summer maize-winter wheat rotation field with a slope of 10° between May 2008 and July 2012. Soil physical and chemical properties (0–20 cm) were evaluated at the start of the field experiment (Table 1). The experiment employed four treatments that combined tillage and crop straw returning practices: 1) soils were ploughed before sowing each crop and crop straws were removed (CT); 2) soils were ploughed before sowing each crop and crop straws were chopped into pieces and returned to the field (CTSR); 3) soils were only ploughed before winter wheat sowing and crop straws were removed from the field (RT); and 4) soils were only ploughed before winter wheat sowing and crop straws were chopped into pieces and returned to the field (RTSR). Regarding tillage, soils were ploughed approximately 2–3 days before sowing. Regarding straw returning, maize residues were chopped into pieces (ca. 5 cm length) and incorporated into the soils when the field was ploughed before winter wheat sowing, while wheat residues were chopped into pieces and left on the soil surface as mulch. The treatments were applied to 36 m^2^ (4 × 9 m) plots arranged in a sequential block design, with three replicates per treatment. A total of twelve plots - separated by concrete borders reaching a depth of 60 cm - were set up, with a tank at the base of each plot to collect runoff. Both maize and wheat were sown in rows that were perpendicular to the slope. Maize seeds were sown with 0.3 m × 0.6 m spacing while wheat was sown with 0.3 m between-row spacing.

In each plot, crops received the same amount of chemical fertilizers (Table 2); the amount of fertilizer followed a nutrient management plan that is common in the region. Regarding the winter wheat season, basal fertilizer was first distributed over the soil, which was then turned over by plowing to transfer the fertilizer to the subsurface (Table 2). Topdressing nitrogen, in the form of urea, was added at the jointing stage. Regarding the summer maize season, basal fertilizer was applied into the furrows after sowing using a hole-sowing machine. Nitrogen topdressing – done at the jointing and tasseling stages – was performed with a hoe so that the N would be incorporated into the soil near the seedlings.

**Table 2:** Annual amount of N, P, K fertilizer (g m^−2^) applied during both winter wheat and summer maize seasons.

Fertilization	Winter wheat season	Summer maize season
Base fertilizer	6 g N m^−2^ (Urea);	11 g N m^−2^ (Urea);
	9 g P_2_O_5_ m^−2^ (Calcium superphosphate)	9 g P_2_O_5_ m^−2^ (Calcium superphosphate)
	6 g K_2_O m^−2^ (Potassium sulfate)	

Topdressing	3 g N m^−2^ (Urea) at the jointing stage	6 g N m^−2^ (Urea) at the jointing stage
		8 g N m^−2^ (Urea) at the tasseling stage

### 2.2 Sampling and measurements

Soil surface runoff was measured in all of the plots during the whole experimental period using the runoff tank collection method. After each rainfall event, the depth of water in the tank was recorded. Water content in the upper 10 cm soil layer was determined volumetrically - at five points in each plot approximately every 30 days - using a TDR probe.

At harvest, crops were manually harvested and threshed. The grains were then air-dried. Furthermore, five soil cores were randomly collected from the upper 10 cm soil layer in each plot using a soil borer. The soil cores collected for each plot were mixed, after which subsamples were air-dried and run through a 100 or 200 mesh sieve to estimate soil organic carbon (SOC), total N (TN), nitrate and ammonium. SOC was measured using the K_2_Cr_2_O_7_ oxidation method (Nelson and Sommers, 1982), while TN was determined using the Kjeldhal method (Bremner, 1996). Soil NO_3_^−^-N and NH_4_^+^-N were extracted with 50 mL of 1 mol L^−1^ KCl (equal to 5.0 g of air-dried soil, 10:1 KCl solution/soil) and analyzed with an automatic flow analyzer (FIAstar 5000 Flow Injection Analyzer; Foss, Hilleroed, Denmark) (Bao, 2000).

Historic daily meteorological data representing the simulation period were obtained from the China meteorological sharing service system (http://cdc.cma.gov.cn/) as well as from a collocated automatic weather station in Xiaofuling (which could only provide data from 2008). Data obtained from these two weather stations can be considered representative of the area because both stations are located close to the experimental site.

### 2.3 Model description and evaluation

A detailed description of the SPACSYS model (https://www.rothamsted.ac.uk/rothamsted-spacsys-model) has been provided in previous studies (Wu et al., 2007; Perego et al., 2016; Li et al., 2017a, 2018a; Zhang et al., 2018). For this reason, only a brief summary of how the model was applied to the current study is presented here. The SPACSYS model includes functions that detail plant phenological development, assimilation, respiration, water and N uptake, partitioning of assimilated N, N fixation for legumes, and root growth and development; these functions can be described with either a one- or three-dimensional root system to predict crop growth and development. Furthermore, the model can be used to accurately predict GHG (e.g. CO_2_ and N_2_O) emissions because it takes into account the organic matter decomposition, mineralization, nitrification and denitrification processes related to C and N cycling.

The model parameterization and evaluation were shown in supplementary data. We evaluated the ability of the SPACSYS model to simulate crop growth and soil nutrient processes in the Danjiangkou Reservoir area, China with data collected from field experiments performed between 2008 and 2012. We further applied the model to assess how reducing tillage intensity and crop straw returning influence soil C and N dynamics and hydrological processes in terms of soil water, C and N balances.

### 2.4 Statistical analyses

The effects of the treatments on the measured or simulated parameters were evaluated by two-way analysis of variance (ANOVA). The least significant difference (LSD) test was used for comparing between-treatment differences in means according to Duncan’s new multiple range tests 0.05 probability level. Figures were made using SigmaPlot 12.0 software.

## 3 Results

### 3.1 Effects of tillage and straw management practices on crop yield

Maize yield was remarkably affected by straw returning while no significant tillage effect, inter-annual variability and interaction effects were observed across the 4-year study (Table 3). RTSR treatment achieved the highest grain yield in 3 out of 4 experimental years, followed by CTSR, while the two practices without straw returning, i.e. CT and RT, were generally the lowest.

**Table 3:** Grain yields (t hm^−2^) of maize and wheat under the CT, CTSR, RT and RTSR treatments over the experimental period.

Year	Maize yield (t ha^−1^)				Wheat yield (t ha^−1^)			
	CT	CTSR	RT	RTSR	CT	CTSR	RT	RTSR
2008-2009	7.52 b	9.26 a	7.96 b	7.82 b	4.72 b	5.11 ab	5.53 a	5.24 ab
2009-2010	9.08 a	8.95 a	7.78 b	9.89 a	5.99 a	5.86 a	5.24 b	5.54 ab
2010-2011	7.59 b	8.78 a	6.78 b	9.10 a	4.12 b	5.17 a	3.68 b	4.86 a
2011-2012	7.13 c	8.03 b	7.00 c	9.39 a	3.46 a	2.97 b	3.38 a	3.29 a
Mean	7.83 b	8.75 a	7.38 b	9.05 a	4.57 a	4.78 a	4.46 a	4.73 a
Multi-way ANOVA								
Tillage (T)	F = 0.05 P = 0.815				F = 0.21 P = 0.650			
Straw returning (S)	F = 15.17 **P< 0.001**				F = 1.85 P = 0.183			
Year (Y)	F = 1.90 P = 0.149				F = 34.59 **P** < **0.001**			
T × S	F = 1.25 P = 0.272				F = 0.04 P = 0.843			
T × Y	F = 0.52 P = 0.670				F = 1.75 P = 0.177			
S × Y	F = 0.51 P = 0.682				F = 2.99 **P** < **0.05**			
T × S × Y	F = 1.83 P = 0.161				F = 0.54 P = 0.656			

Means within a row for Maize and wheat yield separately that are followed by the same letter are not significantly different at *p* < 0.05. Bold numbers mean statistically significant.

By contrast, wheat yield showed a high inter-annual variability, but was not significantly influenced by tillage and straw returning practices. Over the 4-year period, a clear improvement in wheat yield by straw returning was only observed in the year 2010–2011, leading to significant S × Y interaction. Averaged over the 4-year study, 4 treatments were similar in the mean wheat yield, ranging from 4.46 to 4.78 t ha^−1^.

### 3.2 Effects of tillage and straw management practices on surface runoff and soil water content

**Fig. 2:**
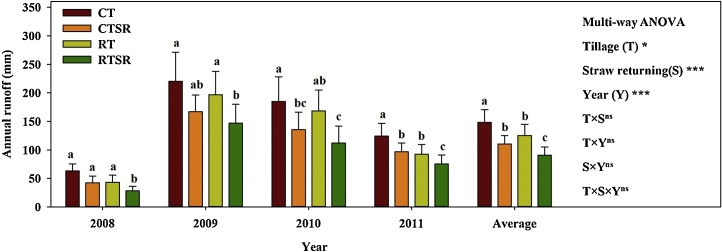
The annual surface runoff under the CT, CTSR, RT and RTSR treatments (2008–2011). Different lowercase letters above the bars in the same year indicate significant between-treatment differences (*p* <  0.05).

As shown in Fig. 2, the amount of annual runoff showed high variations between experimental years, and both tillage and straw management practices significantly influenced annual runoff over the study period. However, no significant T × S, T × Y, S × Y and T × S × Y interactions were observed in the present study. CT treatment generated consistently the highest annual runoff, despite of huge variation between experimental years. The second highest annual runoff occurred in RT treatment, which was slightly lower than in CT treatment. By contrast, the treatments with straw returning, i.e. CTSR and RTSR, substantially decreased the amount of annual runoff than in the corresponding straw removal plots. Averaged over all experimental years, the mean annual runoff was the highest in CT treatment (148 ± 69 mm), followed by that in RT (125 ± 70 mm), while CTSR (110 ± 53 mm) and RTSR(90 ± 50 mm) were the lowest.

**Fig. 3:**
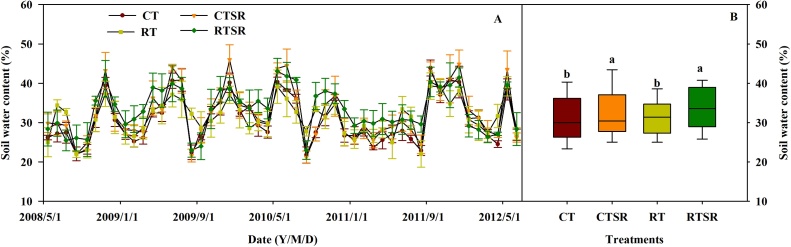
Seasonal variation and mean soil water content (%) in the 0–10 cm soil profile under the CT, CTSR, RT and RTSR treatments (2008–2012). Different lowercase letters above the boxes in the mean indicate significant between-treatment differences (*p* < 0.05).

Soil water content in the 0–10 cm soil depth determined every month showed a high variability between measurements during the study period (Fig. 3A), which generally followed a similar pattern with precipitation. Across the entire period, reduced tillage did not show a significant effect on the mean soil water content, while straw returning significantly increased soil water content in the 0–10 cm soil depth (Fig. 3B).

### 3.3 Effects of tillage and straw management practices on soil carbon and nitrogen content

**Fig. 4:**
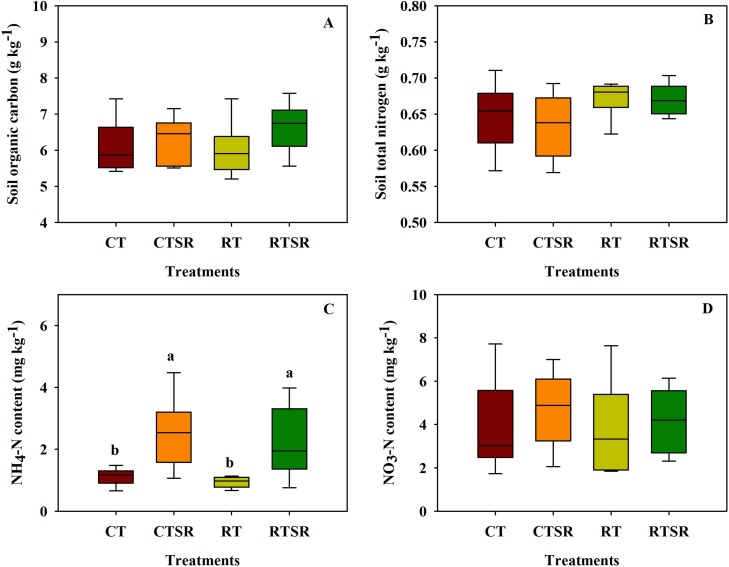
Mean SOC (g kg^−1^), TN (g kg^−1^), NH_4_-N and NO_3_-N (mg kg^−1^) contents under the CT, CTSR, RT and RTSR treatments (2008–2011). Different lowercase letters above the boxes in the mean indicate significant between-treatment differences (*p* <  0.05).

Four soil variables including SOC, total N, NH_4_^+^-N and NO_3_^−^-N were determined after each maize and wheat harvest (Fig. 4). No significant variation in SOC, total N and NO_3_^−^-N was found among the four treatments with different tillage and straw management practices. Straw returning substantially increased soil NH_4_^+^-N content compared to the straw removal treatments, but no significant tillage effect could be observed.

### 3.4 Soil water, carbon and nitrogen balances

The effects of tillage and straw management on soil water, C and N balances were evaluated by means of SPACSYS simulation (for details see the supplementary data). The resultant statistical analysis revealed that the simulations matched the measured data reasonably well, suggesting the SPACSYS model could be applied to assess how different management practices impact soil water, C and N balances.

**Fig. 5:**
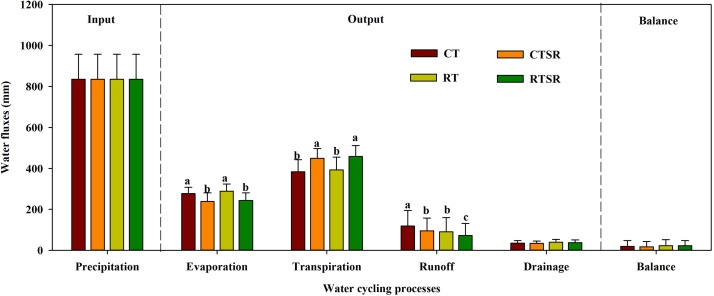
Predicted annual water balance (mm) (0–10 cm soil depth) of the soil-plant system between 2008 and 2018 under the CT, CTSR, RT and RTSR treatments.

The analysis of soil water balance based on the model simulations showed that soil water input almost equaled the output, with slight surpluses ranging from 18 to 23 mm year^−1^ under different tillage and straw management practices (Fig. 5). Soils under different treatments received the same amount of water input (precipitation 835 mm year^-1^). No significant tillage effects could be detected on the key soil water processes including evaporation, transpiration, and drainage. Compared with straw removal treatments (CT and RT), straw returning significantly reduced soil water evaporation and surface runoff, but such effects were somehow offset by enhanced transpiration. Therefore, different tillage and straw returning treatments did not differ in soil water balance.

**Fig. 6:**
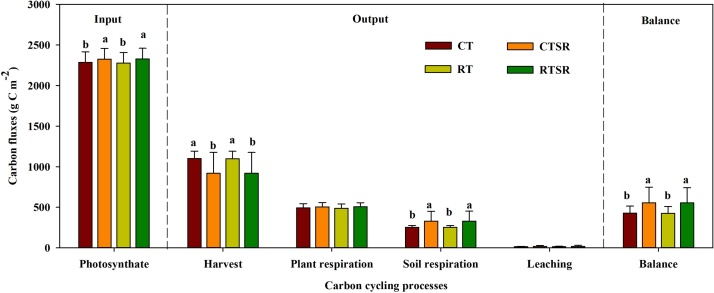
Predicted annual carbon balance (g C m^−2^) (0–10 cm soil depth) of the soil-plant system between 2008 and 2018 under the CT, CTSR, RT and RTSR treatments.

Soils subject to different tillage and straw management practices all performed as a sink for C in the present study (Fig. 6). However, it should be noted that straw returning practices had considerably higher soil C balance than the straw removal treatments. This could be explained mainly by the enhanced photosynthate input and reduced C output by harvest, although straw returning seemed to induce slightly higher C losses through soil respiration. No significant tillage effects were found on soil C cycling processes.

**Fig. 7:**
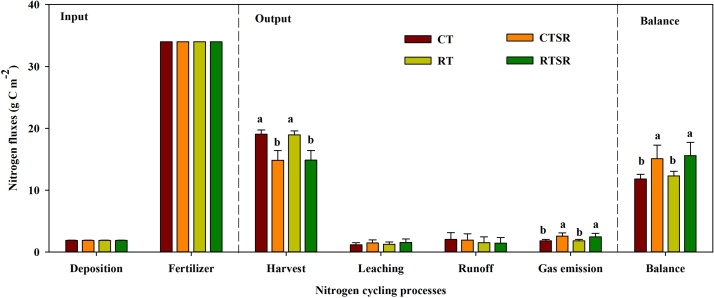
Predicted annual nitrogen balance (g N m^−2^) (0–10 cm soil depth) of the soil-plant system between 2008 and 2018 under the CT, CTSR, RT and RTSR treatments.

All soils under different treatments had net gains of N over the entire simulation period, for which the soil N gains in straw returning practices were substantially higher than in straw removal treatments (Fig. 7). The increased soil N balance by straw returning was mainly due to considerably reduced N output by harvest, despite the slightly increased soil N losses through gas emission. However, reduced tillage did not differ with conventional tillage in soil N fluxes and total N balance.

## 4 Discussion

### 4.1 Tillage and straw management practice effects on crop yield

Conservation farming practices including reduced tillage and straw returning were widely implemented in agroecosystems as a means to maintain agricultural sustainability at lower environmental costs (van Kessel et al., 2013; Busari et al., 2015; Zhang et al., 2015; Tan et al., 2019). The impacts of these practices on crop yields, however, have been variable with decreases, increases and little changes in crop yields reported (Berner et al., 2008; van Kessel et al., 2013; Xu et al., 2019). The mean annual crop yield of 4 maize-wheat rotation cycles ranged from 11.84 to 13.78 t ha^−1^ in the present study (Table 3), which was comparable to those reported by Liu et al. (2017), but was much lower than those of Tan et al. (2019), who revealed an average annual yield of 14.0–18.3 in a two-year winter-maize rotation in Northern China Plain (Tan et al., 2019). This might be explained by the relatively lower indigenous soil fertility and lack of irrigation in the present study region. In agreement with previous studies (Tan et al., 2019; Xu et al., 2019), straw returning significantly enhanced maize yield in this study. The maize yield increase was partly attributed increased soil water availability, as straw returning practices reduced soil water losses through evaporation and surface runoff to ensure efficient crop water uptake and utilization (Fig. 5) (Shao et al., 2016). Moreover, straw returning improved soil fertility (Fig. 4), which favored maize growth and in turn contributed to higher yield. In contrast, no significant straw management effect was observed on wheat yield. This might be attributed to the fact that the benefit effects of straw returning were somewhat offset by the impacts on wheat germination and growth, when the straw decomposed much slower during wheat season with lower soil temperature.

Previous studies suggest that reduced tillage or no tillage can significantly increase crop yield which might be attributed to more efficient conservation and use of soil water and improved soil SOC storage (Fuentes et al., 2003; Xu et al., 2019). However, the reduced tillage treatments did not increase crop yield in the present study (Table 3). Zhang et al. (2013) also observed no NT/RT vs. CT differences in wheat yield in an extensive study comprising 39 sets of experiments spanning 20 years conducted in the dryland area of Loess Plateau of China. A meta-analysis assessing how soil tillage affecting crop performance in the US (74 studies, (Ogle et al., 2012)) reported that crop productivity could be reduced with the adoption of RT/NT in cooler and/or wetter climatic conditions whereas yields increased in the drier climate zones. van Kessel et al. (2013) found further that crop yield responses to RT/NT may depend on both climate condition and experimental duration. Therefore, field experiments with long duration are need to better elucidate the impacts of conservation tillage on crop yield under different climate conditions before site-specific adoption of these practices.

### 4.2 Tillage and straw management practice effects on water balance

Water that enters the soil and plant systems is partitioned to soil evaporation, transpiration, surface runoff loss, water fluxes into drainage and soil water storage. Evapotranspiration (ET), which comprises both soil-surface evaporation and plant transpiration, is a major component of the water balance in the systems (Tan et al., 2002). In the present study, the straw returning treatments significantly improved crop yields when compared to the CT treatment, and straw returning brought about reduces of water evaporation losses, together with increases of transpiration. One explanation for this finding is that straw returning improves the microclimate and strongly reduces water exchange from the soil to the air by promoting plant transpiration at the expense of evaporation from the soil (Dong et al., 2018); this dynamic may have fostered the observed biomass accumulation.

Moreover, the model simulations showed that both straw returning and reduced tillage practices decreased water loss by surface runoff. Previous studies have shown that straw returning and conservation tillage to reduce runoff should be based on different mechanisms (Li et al., 2011; Liu et al., 2012; Ram et al., 2012). Maintaining sufficient water absorption capability during rainfall, which will generate runoff, is also important since straw provides additional pathways for water infiltration (Ram et al., 2012). Generally, straw returning promotes water infiltration because the addition of soil organic matter modifies soil physical properties (Tan et al., 2002; Liu et al., 2012). Thus, the straw returning treatments appear to have allowed the trapped water to remain in the straw for longer periods of time; this will decrease the velocity of surface flow and increase infiltration (Li et al., 2011). Furthermore, treatments that included reduced tillage practices also significantly decreased soil surface runoff when compared to the CT treatment. Reduced tillage, which meant that plots not ploughed during the maize sowing, did not destroy the hedge structure formed by wheat roots and stubble; as a result, this hedge structure acted as a permeable barrier to effectively slow down the runoff flow rate (Wang et al., 2010), which, in turn, reduced runoff losses.

### 4.3 Tillage and straw management practice effects on carbon and nitrogen balance

Agro-ecosystems have recently been in the research spotlight due to their role in climate change; more specifically, farmlands, which are an example of how humans modify the natural environment, have the potential to largely affect C exchange between soils and the atmosphere (Camarotto et al., 2018). Various field management practices, such as tillage, fertilization and irrigation, have direct and/or indirect implications for the soil C balance (Zhang et al., 2018). Our simulations showed that soils in all four tested treatments acted as a C sink. The most likely reason is that the plots had low initial soil organic matter content, which translates to the soils having higher C storage potential (Kucerik et al., 2018). The simulation also demonstrated that straw returning significantly promotes SOC accumulation, with the primary mechanism being the enhancement of photosynthetic C input. Furthermore, straw returning remarkably accelerated soil respiration (Fig. 6), which was consistent with previous studies (Wu et al., 2017). This is probably due to the enhanced soil organic catabolism of the straw, and relatively high soil respiration substrates (Yang et al., 2018).

Nitrogen loss seemed to be most strongly related to soil water loss through leaching and surface runoff. An explanation for this finding is that N loss is directly linked to the amount of soil water loss and N concentration in water (Wang et al., 2015). For example, simulations showed that the increase in soil N content would enhance N leaching in both the CTSR and RTSR treatments. N runoff loss was 10–33% lower in plots that included straw returning and reduced tillage treatments when compared to CT plots. The reduction of N loss from runoff imparted by residue retention could be explained by the associated decrease in surface runoff, which was caused by increased surface roughness and water absorption capacity (Franklin et al., 2007). The performed simulations also showed that straw returning increased N loss through gas emission, which might be explained by increased soil nutrient contents and biological activity. This result is consistent with the findings reported in previous studies of flat fields (Zhang et al., 2018).

As expected, both observations and simulations demonstrated that reduced tillage with straw returning led to increases in grain yield with lower C and N losses. Soil water redistribution in soil, and C and N cycling are controlled by field management practices, especially crop types, fertilizer application, tillage and straw returning (Li et al., 2011; Ram et al., 2012). Such an approach can provide public goods in the forms of higher yields, enhanced soil quality, and reduced agricultural non-point source pollution and GHG emissions (Chen et al., 2011; Wu et al., 2016). However, there might be other alternative practices to achieve the above objectives (Zhao et al., 2012). Therefore, future studies need to focus on identifying more field management strategies (such as fertilization, crop rotation and layout) using the modelling approach.

## 5 Conclusions

Maize yield was remarkably affected by straw returning while no significant tillage effect. By contrast, wheat yield showed a high inter-annual variability, but was not significantly influenced by tillage and straw returning practices. The soil water balance analysis demonstrated that the implementation of straw returning reduced water loss relative to treatments without straw returning mainly through decreases in evaporation and runoff, but increased transpiration. The simulations showed that all of the treatments acted as C and N sinks, and that treatments with straw returning (CTSR and RTSR) amassed more C and N in the soil than those without straw returning (CT and RT). The results demonstrate that in maize-wheat rotation slopping land reduced tillage with straw returning is a win-win practice for the equilibrium between agricultural productivity and low soil water, C and N losses.
