# Supplementary material for: Soil C and N dynamics and hydrological processes in a maize-wheat rotation field subjected to different tillage and straw management practices
Source: Agric Ecosyst Environ. 2019 Dec 1;285:106616. doi: 10.1016/j.agee.2019.106616 (PMC6743210; doi:10.1016/j.agee.2019.106616)
Supplement: Supplementary file 1 [file mmc1.doc]

**Supplementary Data: The SPACSYS model parametrization and evaluation**

We evaluated the ability of the SPACSYS model to simulate soil C and N dynamics and hydrological processes in terms of crop yield, soil surface runoff, soil water, soil organic C, total N, as well as nitrate and ammonium contents under different tillage and straw management practices with data collected from field experiments performed between 2008 and 2012 in the Danjiangkou Reservoir area, China.

The optimized key parameters about the processes of water movement and soil C and N cycling were shown in Table S1. The following diagnostics were used to evaluate model performance by comparing the SPACSYS simulated data with the measured data: (i) the correlation coefficient (r), (ii) the modelling efficiency (EF), (iii) the root mean square error (RMSE), (iv) the coefficient of determination (CD), (v) the relative error (RE), (vi) the maximum error (ME) (Table S2).

A comparison of the measured and simulated maize and wheat yields is shown in Table S3. And the comparisons between simulated and observed surface runoff is shown in Fig. S1. The simulated and observed dynamics of soil water content, SOC, TN, nitrate and ammonium in the top 10cm soil layer are shown in Fig. S2 and S3.

In conclusion, the SPACSYS model is a promising tool for analyzing soil C and N dynamics and hydrological processes in maize-wheat rotation systems subjected to different tillage and straw management practices.

**Table S1** Optimized key parameters related to soil-plant water processes and soil C and N cycles in SPACSYS.

| **Parameter** | **Value** | **Unit** |
| --- | --- | --- |
| ***Soil-plant water process parameters*** |  |  |
| Canopy albedo | 23 | % |
| Albedo for dry soil | 30 | % |
| Albedo for wet soil | 15 | % |
| Coefficient in albedo curve | 1 | - |
| Correspond amount cover surface | 50 | mm |
| Distance between drainpipes | 10 | m |
| Interception storage capacity per LAI | 0.5 | mm LAI^-1^ |
| Minimum roughness length | 0.01 | m |
| Maximum water amount stored on the soil surface without causing any surface runoff surface | 10 | mm |
| Maximum canopy density | 0.7 | - |
| Minimum hydraulic conductivity | 0.0 | mm d^-1^ |
| Runoff first order rate coefficient | 0.3 | d^-1^ |
| Sensitivity to water tension | 100 | - |
| Surface resistance when intercepted water occurs | 5.0 | s m^-1^ |
| ***Soil C and N cycle parameters*** |  |  |
| Coefficient in water function | 0.6 | - |
| Dry deposition mineral N to soil surface | 0.004 | g N m^-2^ d^-1^ |
| Humus fraction from dissolved | 0.245 | - |
| Humus fraction from fresh litter | 0.01 | - |
| Humus fraction from fresh organic matter | 0.01 | - |
| Potential decomposition rate for humus | 0.0434 | d^-1^ |
| Critical C/N ratio favorable to immobilization | 10 | - |
| Microbial maintenance respiration rate | 0.4 | - |
| Assimilation factor | 0.4 | - |
| Fraction of ammonium content for immobilization | 0.6 | - |
| Base temp to unity mineralization | 13 | °C |
| Base temp to unity nitrification | 13 | °C |
| Base temperature to unity denitrification | 27 | °C |
| Q10 value for denitrification | 2.5 | - |
| Q10 value for mineralization | 2.5 | - |
| Q10 value for nitrification | 1.23 | - |
| C N ratio of microbes | 10.0 | - |

**Table S2** Statistical analyses of SPACSYS model performance in terms of yield, soil surface runoff, soil water content and SOC TN, NH_4_-N and NO_3_-N contents.

| **Index** | **Pairs of samples** | **r** | **EF** | **RMSE (%)** | **CD** | **RE** | **ME** |
| --- | --- | --- | --- | --- | --- | --- | --- |
| **Maize yield** | 16 | 0.79 | 0.50 | 7.90 | 3.9 | -2.6 | 1.0 |
| **Wheat yield** | 16 | 0.93 | 0.66 | 12.10 | 0.9 | -10.2 | 1.4 |
| **Surface runoff** | 264 | 0.74 | 0.47 | 61.18 | 1.4 | 19.5 | 17.3 |
| **Soil water content** | 248 | 0.88 | 0.52 | 12.70 | 0.8 | 8.8 | 11.1 |
| **SOC content** | 36 | 0.43 | 0.16 | 9.70 | 8.4 | 0.2 | 1.2 |
| **TN content** | 36 | 0.51 | 0.14 | 2.10 | 26.3 | -0.3 | 0.0 |
| **NH_4_-N content** | 36 | 0.97 | 0.93 | 15.90 | 1.3 | -2.2 | 0.6 |
| **NO_3_-N content** | 36 | 0.70 | 0.37 | 25.00 | 1.0 | 3.6 | 1.3 |

r is the correlation coefficient between the measured and simulated data; EF is modelling efficiency; RMSE is root mean squared error; CD is coefficient of determination; RE is relative error; ME is mean error.

**Table S3 Simulated (S) and measured (M) grain yields (t hm^-2^) of maize and wheat under the CT, CTSR, RT and RTSR treatments over the experimental period.**

| **Crop** | **Year** | **CT** |  | **CTSR** |  | **RT** |  | **RTSR** |  |
| --- | --- | --- | --- | --- | --- | --- | --- | --- | --- |
|  |  | S | M | S | M | S | M | S | M |
| **Maize** | 2008 | 8.4 | 7.5±0.8 | 8.3 | 9.3±1.5 | 8.5 | 8.0±0.5 | 8.4 | 7.8±1.3 |
|  | 2009 | 8.4 | 9.1±0.6 | 9.1 | 9.0±1.2 | 8.5 | 7.8±0.8 | 9.2 | 9.9±0.6 |
|  | 2010 | 8.1 | 7.6±0.4 | 8.7 | 8.8±1.1 | 7.8 | 6.8±1.4 | 8.7 | 9.1±1.7 |
|  | 2011 | 7.5 | 7.1±0.8 | 8.6 | 8.0±1.0 | 7.7 | 7.0±0.9 | 8.6 | 9.4±2.2 |
|  | **Average** | **8.1** | **7.8±0.9** | **8.7** | **8.8±0.5** | **8.1** | **7.4±0.6** | **8.7** | **9.0±0.9** |
| **Wheat** | 2009 | 5.5 | 4.7±0.6 | 5.5 | 5.1±0.6 | 5.5 | 5.5±0.5 | 5.5 | 5.2±0.7 |
|  | 2010 | 6.0 | 6.0±0.7 | 6.1 | 5.9±0.6 | 6.0 | 5.2±0.4 | 6.1 | 5.5±1.2 |
|  | 2011 | 5.0 | 4.1±0.4 | 5.3 | 5.2±0.7 | 5.0 | 3.7±0.5 | 5.3 | 4.9±0.8 |
|  | 2012 | 3.6 | 3.5±0.4 | 3.6 | 3.0±0.4 | 3.5 | 3.4±0.3 | 3.6 | 3.3±0.3 |
|  | **Average** | **5.0** | **4.6±1.1** | **5.1** | **4.8±1.3** | **5.0** | **4.5±1.1** | **5.1** | **4.7±1.0** |


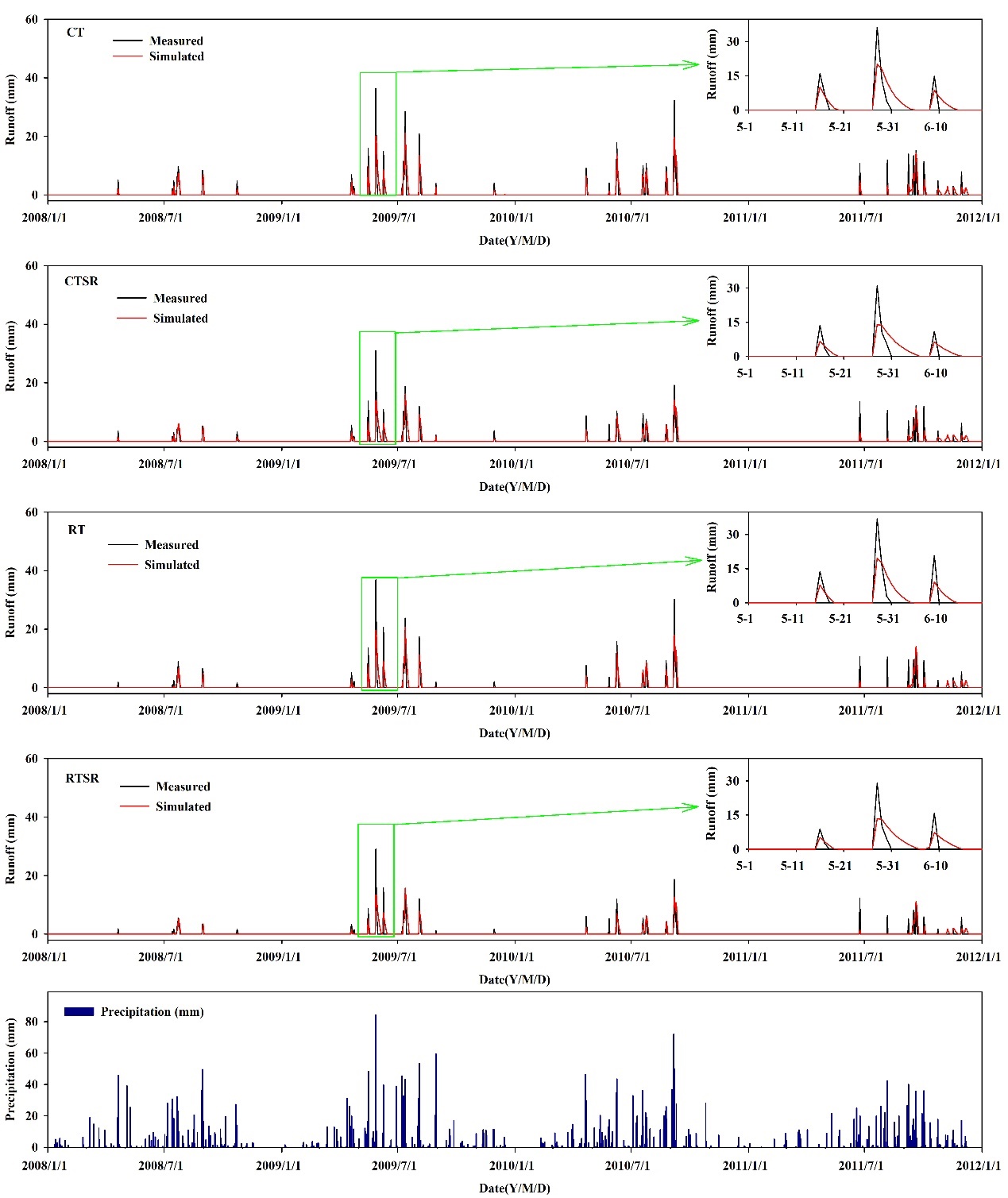


**Fig. S1** Comparisons of simulated and measured soil surface runoff, along with total precipitation over the study period, under the CT, CTSR, RT and RTSR treatments. The insets illustrate the detail comparison of simulated and measured water flow after high rainfall events.


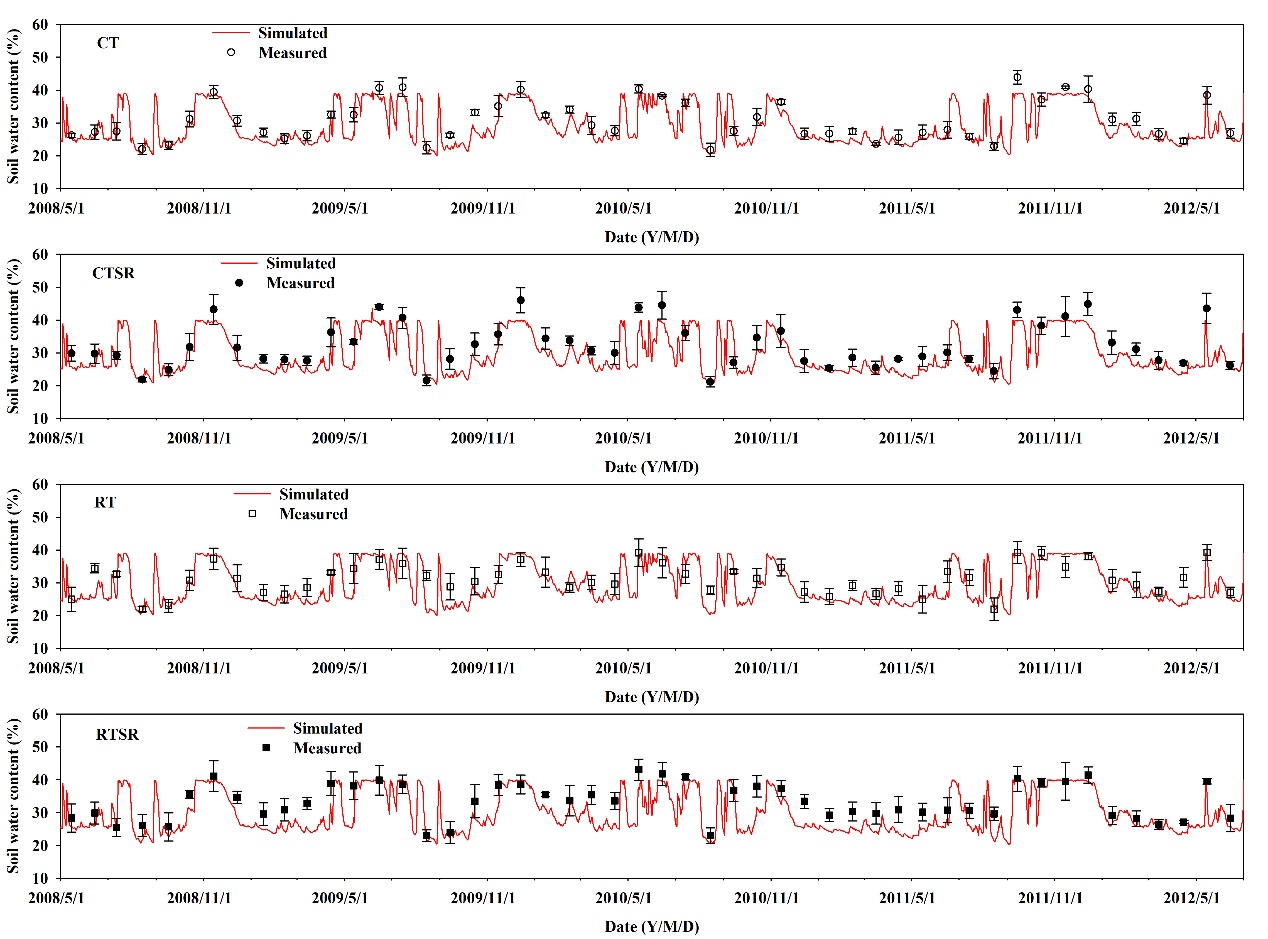


**Fig. S2** Temporal comparison of measured (points) and simulated (lines) soil water contents (%) under the CT, CTSR, RT and RTSR treatments. The error bars represent standard deviations of data points (n = 3).


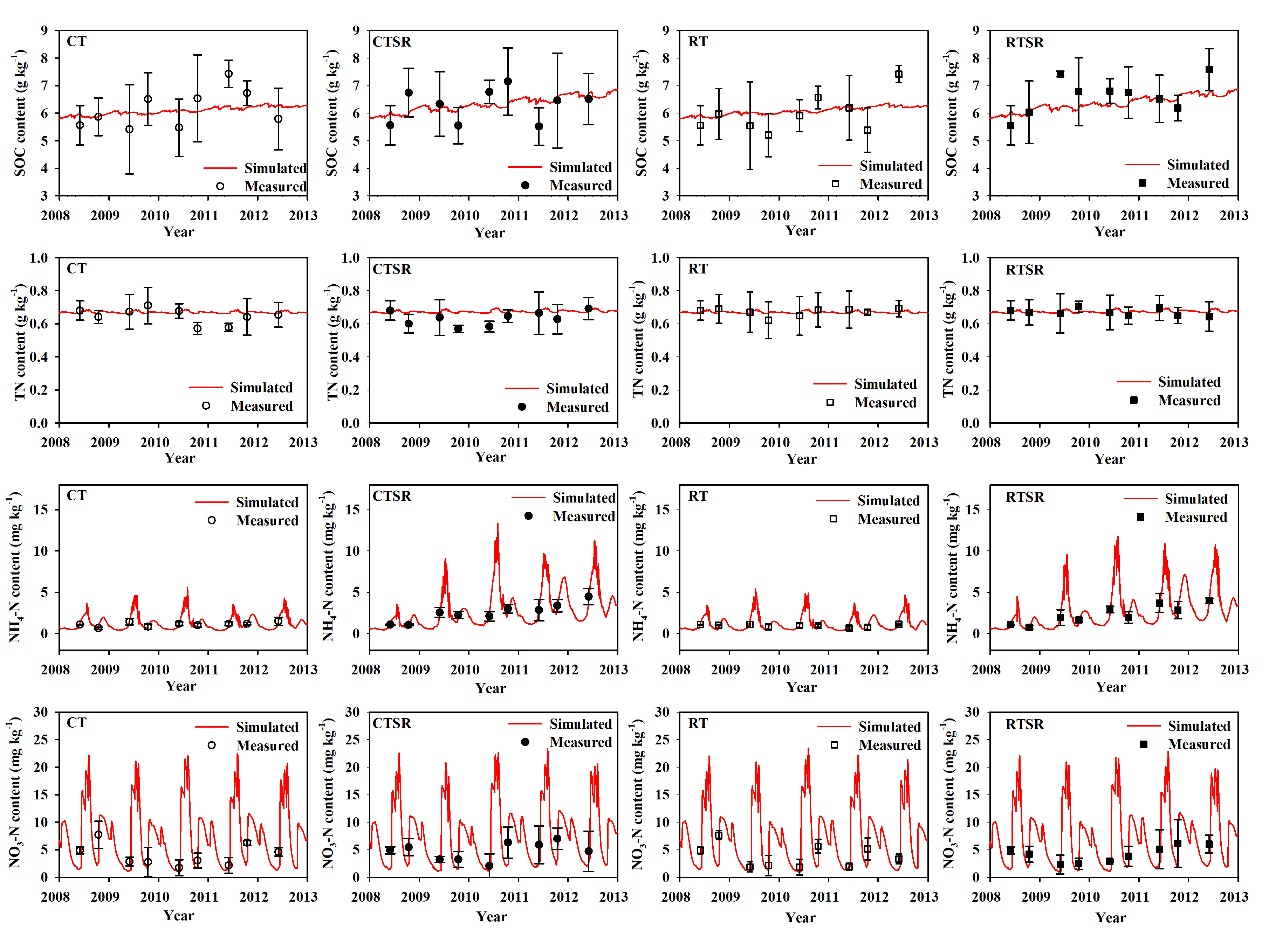


**Fig. S3** Temporal comparison of measured (points) and simulated (lines) SOC (g kg^-1^), TN (g kg^-1^), NH_4_-N and NO_3_-N (mg kg^-1^) contents under the CT, CTSR, RT and RTSR treatments. The error bars represent standard deviations of data points (n = 3)
